# Anandamide modulates WNT-5A/BCL-2, IP3/NFATc1, and HMGB1/NF-κB trajectories to protect against mercuric chloride-induced acute kidney injury

**DOI:** 10.1038/s41598-023-38659-9

**Published:** 2023-07-24

**Authors:** Dalaal M. Abdallah, Mahmoud M. Kamal, Nour Eldin S. Aly, Hanan S. El-Abhar

**Affiliations:** 1 Pharmacology and Toxicology Department, Faculty of Pharmacy, Cairo University, Cairo, 11562 Egypt; 2 Research Institute of Medical Entomology, General Organization for Teaching Hospitals and Institutes, Cairo, Egypt; 3 Department of Pharmacology, Toxicology, and Biochemistry, Faculty of Pharmacy, Future University in Egypt (FUE), Cairo, 11835 Egypt

**Keywords:** Diseases, Nephrology

## Abstract

Endocannabinoid anandamide (AEA) has a physiological role in regulating renal blood flow, whereas its analogs ameliorated renal ischemia/reperfusion injury. Nonetheless, the role of AEA against mercuric chloride (HgCl_2_)-induced renal toxicity has not been unraveled. Rats were allocated into control, HgCl_2_, and HgCl_2_/AEA treated groups. The administration of AEA quelled the HgCl_2_-mediated increase in inositol trisphosphate (IP3) and nuclear factor of activated T-cells cytoplasmic 1 (NFATc1). The endocannabinoid also signified its anti-inflammatory potential by turning off the inflammatory cascade evidenced by the suppression of high mobility group box protein-1 (HMGB1), receptor of glycated end products (RAGE), nuclear factor-κB p65 (NF-κB), and unexpectedly PPAR-γ. Additionally, the aptitude of AEA to inhibit malondialdehyde and boost glutathione points to its antioxidant capacity. Moreover, AEA by enhancing the depleted renal WNT-5A and reducing cystatin-C and KIM-1 (two kidney function parameters) partly verified its anti-apoptotic capacity, confirmed by inhibiting caspase-3 and increasing B-cell lymphoma-2 (BCL-2). The beneficial effect of AEA was mirrored by the improved architecture and kidney function evidenced by the reduction in cystatin-C, KIM-1, creatinine, BUN, and caspase1-induced activated IL-18. In conclusion, our results verify the reno-protective potential of AEA against HgCl_2_-induced kidney injury by its anti-inflammatory, antioxidant, and anti-apoptotic capacities by modulating WNT-5A/BCL-2, IP3/NFATC1, HMGB-1/RAGE/NF-κB, caspase-1/IL-18, and caspase-3/BCL-2 cues.

## Introduction

Mercury is the third most toxic environmental pollutant that is never destroyed; hence, imposing a global threat to humans and the ecosystem^1^. Acute inorganic mercurial intoxication is due to exposure to large amounts of mercuric-chloride (HgCl_2_) in the industry and the chronic exposure is related to anthropogenic activity during artisanal small-scale gold mining^2^. The primary target organ for HgCl_2_ toxicity is the kidney, with the proximal tubule S3 segment being the principal target site of toxicity, where it accumulates to trigger injury^3^. By its corrosive/pro-oxidant nature, interaction with cellular sulfhydryl groups, and increasing intracellular calcium (Ca_i_^2+^), inorganic, mercury induces renal injury and apoptosis by interfering with several pathways^3–5^.

Being one culprit of its renal cytotoxic effect, the accumulated Hg stimulates Ca^2+^ channels to permit Ca^2+^ cellular influx an its capacity to injure endoplasmic reticulum (ER) releases the sequestered Ca^2+^ causing its intracellular buildup^5^. The slightest disturbance in Ca^2+^ homeostasis induces cellular disruption by activating different signaling pathways including the phospholipase C (PLC) signal^6^. In a vicious cycle, activated PLC converts phosphatidylinositol 4,5-bisphosphate (PIP2) into diacylglycerol (DAG) and inositol trisphosphate (IP3)^7^, where the latter can further increase the release of Ca_i_^2+^ upon activating its receptor (IP3R). The intracellular accrued Ca^2+^ subsequently activates the calcineurin/nuclear factor of the activated T-cells (NFAT) pathway via dephosphorylating NFAT cytoplasmic 1 (NFATc1) and its nuclear translocation^7,8^. The NFAT family members are considered transcription factors involved in the transcription of several genes, including cytokine genes^8^. A strict role for the activated calcineurin pathway in human podocyte-related nephropathy has been previously documented^9^; however, the involvement of the IP3/NFAT pathway in HgCl_2_ kidney injury has not been studied before.

One of the inflammatory mediators that partake in the pathogenesis of a wide range of kidney diseases is the high mobility group box-1 (HMGB-1), which is a nuclear protein naturally bound to DNA. HMGB-1 initiates the pro-inflammatory responses by activating the receptor for advanced glycation end products (RAGE) and Toll-like receptor 4 (TLR4) following its extracellular release^10^. Consequently, the transcription factor nuclear factor-κB (NF-κB) is activated^11^ to transcribe pro-interleukin-18 (IL-18), which is cleaved by caspase-1 to give the mature inflammatory cytokine that mediates renal injury^12^.

The wingless-related integration site (WNT) family also participates in kidney physiology/pathophysiology^13^ and is divided into the canonical WNT/β-catenin pathway and the non-canonical one, which is further subdivided into the planar cell polarity and WNT/Ca^2+^ pathways. The WNT proteins with their different trajectories are versatile players in kidney healing and injury, based on the activation period^14^. WNT-5A is one of the upstream protein/ligand of the non-canonical signaling pathway. However, its role in kidney injury is debatable, where in different kidney models WNT-5A had a role in fibrosis^15^, inflammation via the WNT-5A/NF-κB axis^16^, and sepsis^17^. Moreover, in an obesity model, WNT-5A promoted inflammation and insulin resistance^18^. Contrariwise, other studies revealed the downregulation of WNT-5A mRNA in *Mycobacterium tuberculosis* aerosol-infected mice^19^ and its ability to increase anti-inflammatory cytokines and inhibit the TLR4/NF-κB inflammatory cascade in a model of LPS-induced macrophage activation, as well as in septic patients^20^.

One of the systems that influence kidney physiology and pathophysiology is the endocannabinoid system, which plays a critical role in renal function as characterized in clinical and experimental settings^21^. In a normal kidney, the two cannabinoid receptors (CBRs) are identified in the medullary and cortical cells, though their expression is very low except for CB2R in the glomeruli and CB1R in the renal vasculature with hemodynamic roles^22,23^. Although some studies reported that under nephropathic conditions, the CB1R is upregulated and CB2R is downregulated^22,24^, the exact role and expression of either receptor in cases of kidney injury are still controversial. For instance, Chafik et al*.*^25^ recently reported that the expression of CB2R was upregulated in a model of cisplatin-induced kidney injury, while other studies documented the overexpression of both CBRs in the proximal tubules during renal injury^26,27^. Besides, earlier data highlighted the injurious role of activated CB1R; for example, two studies stated that activated CB1R has induced apoptosis in primary cultured rat mesangial cells^28^ and renal fibrosis in multiple nephropathies^29^. Inversely, the specific deletion of CB1R attenuated renal dysfunction via hampering lipo-toxicity in the renal proximal tubules of obese mice^30^; and in the same beneficial context its activation afforded renoprotection against an ischemia/reperfusion (I/R) insult^31^. Similarly, the activation of CB2R was reported to promote fibrosis in unilateral I/R and folic acid nephropathy mouse models and renal tubular mitochondrial dysfunction in aged mice^27,32^ but it mediated renoprotection in diabetes, I/R, and cisplatin nephropathy models^25,33–35^.

The first discovered endogenous ligand of the CBRs is anandamide (*N*-arachidonoyl-ethanolamine; AEA), which besides binding CB1R and CB2R, it activates other receptors, such as the peroxisome proliferator-activated receptors (PPARs) and the GPR18/88^36^. Noteworthy, AEA and its metabolizing/degrading enzymes are abundant in the kidney cortex and medulla^24^. In addition to regulating inflammation in l-homocysteine stimulated podocytes, AEA and/or its metabolites mediate vasorelaxant and neuro-modulatory effects, as well as tubular reabsorption of sodium and fluids for long-term blood pressure control^37,38^.

According to the above data, the impact of HgCl_2_-induced kidney injury on some of the previous mentioned signaling pathways, and the potential regulatory effect of AEA on these hubs has not been unveiled. Therefore, here we hypothesized that AEA may exert its reno-protective effect against a model of HgCl_2_-induced AKI via limiting inflammation, oxidative stress, and apoptosis. We assessed the possible role of the non-canonical WNT-5A/BCL-2, IP3/NFATc1, HMGB-1/RAGE/NF-κB/IL-18 trajectories besides PPAR-γ as signaling pathways of the HgCl_2_-induced kidney injury and the modulatory effect of AEA on these hubs.

## Material and methods

### Animals

Adult 8-week-old male Wistar rats (180–220 g) were purchased from the breeding colony at the animal house of the Research Institute of Medical Entomology (RIME, Giza, Egypt). Rats were allowed to acclimatize for a week before experiment was carried out; and they were housed under constant environmental conditions (12 h light/dark cycles, an ambient temperature of 22 ± 2 °C, and a humidity level of 65–70%). Animals were fed normal diet chow with free access to water ad libitum. This study abides the ARRIVE guideline and complies with the Guide for the Care and Use of Laboratory Animals (NIH Publication No. 85-23, revised 2011); and was approved by the Research Ethics Committee of the Faculty of Pharmacy, Cairo University (Cairo, Egypt; PT: 1325).

### Induction of acute nephrotoxicity by HgCl_2_

Nephrotoxicity was induced by a single subcutaneous dose of HgCl_2_ (3 mg/kg; Sigma-Aldrich, MO, USA) dissolved in saline^39^.

### Experimental design

Rats were randomly divided into 3 groups (n = 6 each); in the first group, animals served as the control group, whereas those in the second group received HgCl_2_ and were designated as the nephrotoxic group. Rats in the third group were injected with AEA (2.5 mg/kg; i.p; Tocris Bioscience, Bristol, UK) 30 min before and 24 h after the induction of AKI^40^ (AEA group). Twenty-four hours after the last dose of AEA, animals were anesthetized by a high dose of thiopental (100 mg/kg) and blood was rapidly collected from the femoral vein to prepare sera samples to assess renal function. Afterward, the left kidney of all rats was excised, decapsulated, and homogenized in phosphate buffer saline (PBS) for biochemical assessments. Homogenates were aliquoted for the determination of the intended parameters and stored at − 80 °C. The right kidney of 3 representative rats/group was used for histopathological analysis.

### Biochemical analysis

#### Assessment of serum parameters

Serum creatinine was quantified by a purchased spectrophotometric assay kit (Bioassay Systems, CA, USA; cat# DICT-500) using an improved Jaffe method that utilizes picrate to form a red-colored complex with creatinine. Blood urea nitrogen (BUN) was evaluated spectrophotometrically using a colorimetric assay kit (Bioassay Systems; cat# DIUR-500) that depends on the modified Berthelot reaction. Serum levels of cystatin C (CUSABIO, MD, USA; cat# CSB-E08385r), kidney injury molecule-1 (KIM-1; MyBioSource, CA, USA; cat# MBS2702467), and IL-18 (LSBio, WA, USA; cat# LS-F2596) were determined using the corresponding rat ELISA kits. All experiments were processed according to the manufacturers’ instructions.

#### Assessment of renal parameters

The corresponding rat ELISA kits purchased from CUSABIO were used to determine the renal contents of IP3 (cat# CSB-E13004r), HMGB-1 (cat# CSB-E08224r), and B-cell lymphoma-2 (BCL-2; cat# CSB-E08854r), whereas those from LSBio were obtained to assess PPAR-γ (cat# LS-F4266) and caspase-1 (cat# LS-F6716). Additionally, MyBioSource ELISA kits were purchased for the determination of NFATc1 (cat# MBS7236705) and malondialdehyde (MDA; cat# MBS738685). Besides, ELISA kits for WNT-5A (Abbexa, Cambridge, UK; cat# abx258208), *p*S536-NF-κB p65 (Abcam, Cambridge, UK; cat# ab176647), RAGE (RayBiotech, GA, USA; cat# ELR-RAGE-1), and glutathione (GSH; Amsbio, Abingdon, UK; cat# AMS.E02G0367) were procured from the corresponding companies in parenthesis. All estimates were performed according to the manufacturers' prescripts.

#### Determination of renal caspase-3 activity

The activity of caspase-3 was determined using the colorimetric assay kit (Sigma-Aldrich; cat# CASP3C-1KT) that is based on the hydrolysis of the peptide substrate acetyl-Asp-Glu-Val-Asp p-nitroanilide (Ac-DEVD-pNA) by caspase-3. This results in the release of the p-nitroaniline (pNA) moiety that has a high absorbance at 405 nm (ε^mM^ = 10.5), which is proportional to the enzyme activity.

### Renal histological examination

The harvested right kidneys were kept in 10% formal saline and then embedded in paraffin blocks. Transverse sections of 5 μm thickness were prepared and stained with Haematoxylin and Eosin (H&E) and inspected blindly by a pathologist under a light microscope (BX43, Olympus, Tokyo, Japan) and photographed using the Cellsens dimension software connected to the Olympus DP27 camera. In five non-overlapping microscopic fields per animal (n = 3 each), the collective renal histopathological damage was assessed as the sum of individual lesions representing congestion of glomerular tufts/renal blood vessels (CO), inflammatory cell infiltration (IF), necrosis of tubular epithelium (NC), and renal cast (RC) that were graded/scored from 0 to 5 according to the involved percent of the kidney: 0 = normal; 1 = mild (< 10%); 2 = moderate (10–25%); 3 = severe (25–50%); 4 = very severe (50–75%); 5 = extensive damage (> 75%)^41^.

### Statistical analysis

Parametric data were expressed as means ± SD (n = 6 each). Comparisons between means were carried out using a one-way analysis of variance (ANOVA) followed by the Tukey's Multiple Comparison post hoc test. Non-parametric data are presented as median with range and analyzed using Kruskal Wallis test followed by the post hoc Dunn’s test. A probability level of less than 0.05 was accepted as being significant in the used statistical tests.

## Results

### AEA improves renal function in HgCl_2_-induced AKI

**Figure 1:**
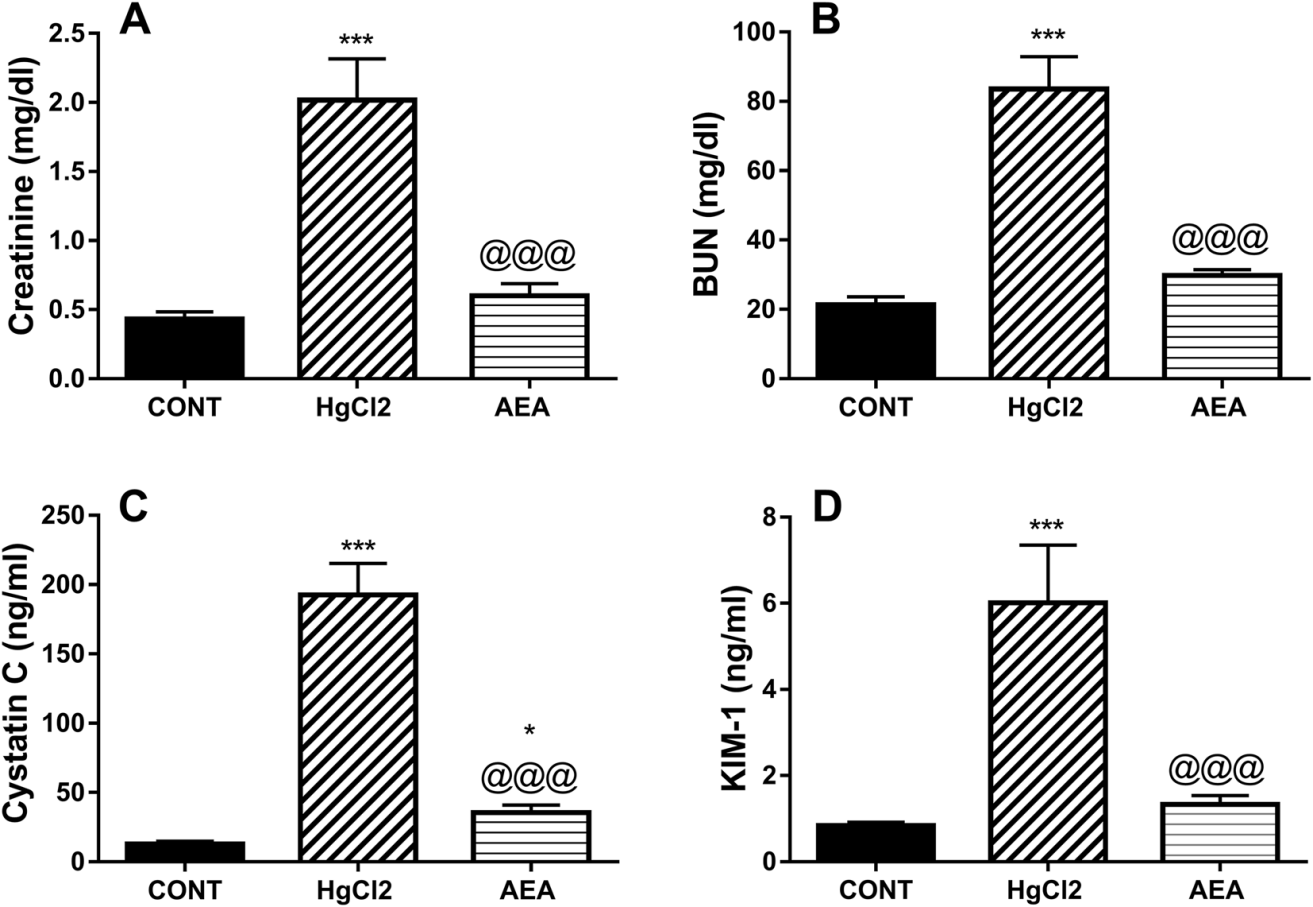
Effect of AEA on serum (**A**) creatinine, (**B**) BUN, (**C**) cystatin-C, and (**D**) KIM-1 in HgCl_2_-induced AKI in rats. Data are expressed as the mean of 6 rats ± SD. *P < 0.05 and ***P < 0.001 compared with CONT and ^@@@^P < 0.001 compared with HgCl_2_ groups using one-way ANOVA followed by Tukey’s Multiple Comparison post hoc test. *AEA* anandamide, *AKI* acute kidney injury, *BUN* blood urea nitrogen, *CONT* control, *HgCl*_*2*_ mercuric chloride, *KIM-1* kidney injury molecule-1.

As depicted in Fig. 1, exposure of animals to HgCl_2_ markedly increased the serum levels of (A) creatinine (1.4 folds), (B) BUN (1.3 folds), (C) cystatin-C (2.4 folds), and (D) KIM-1 (1.6 folds) compared to the control value. On the other hand, the administration of AEA succeeded to bring both serum creatinine and BUN to the normal level and markedly abridged cystatin C by 82% and KIM-1 by 78%, as compared to the insult.

### AEA turns off the IP3/NFATc1 signaling pathway in HgCl_2_-induced AKI

**Figure 2:**
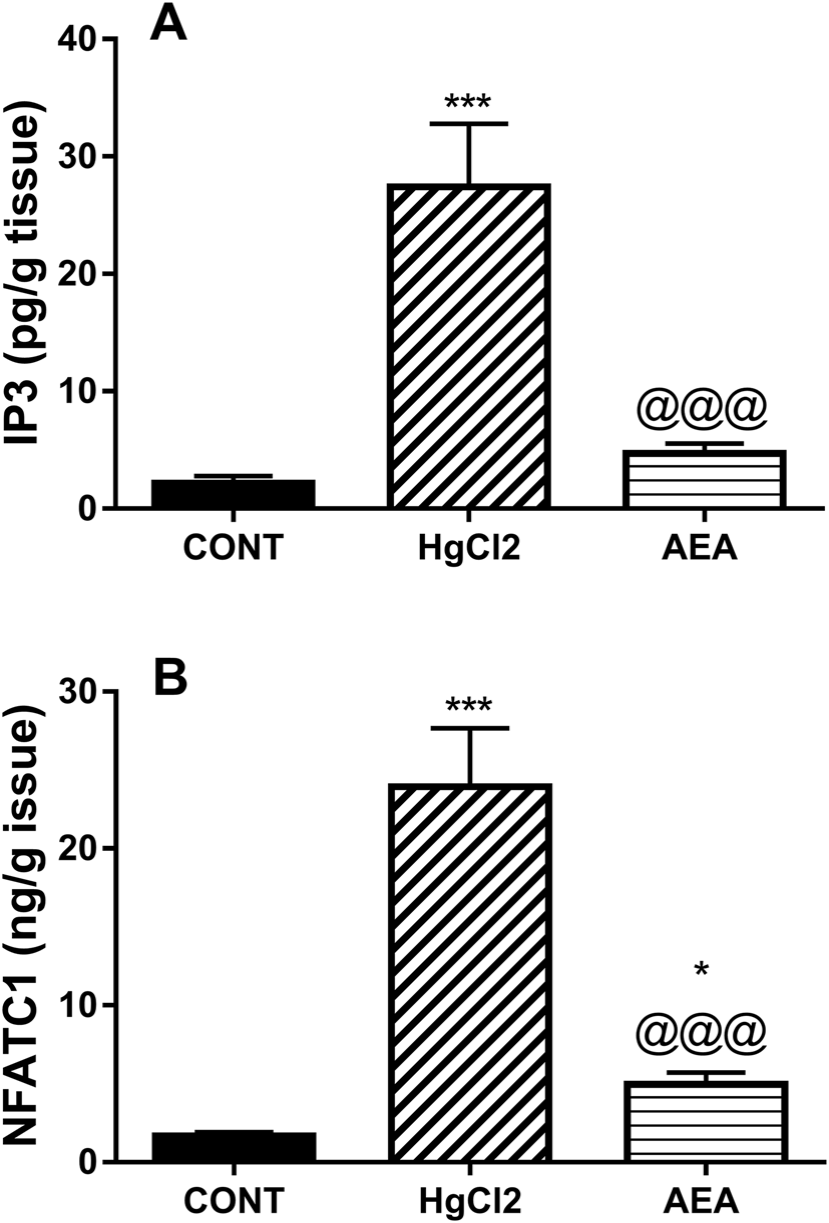
Effect of AEA on renal contents of (**A**) IP3 and (**B**) NFATc1 in HgCl_2_-induced AKI in rats. Data are expressed as the mean of 6 rats ± SD. *P < 0.05 and ***P < 0.001 compared with CONT and ^@@@^P < 0.001 compared with HgCl_2_ groups using one-way ANOVA followed by Tukey's Multiple Comparison post hoc test. *AEA* anandamide, *AKI* acute kidney injury, *CONT* control, *HgCl*_*2*_ mercuric chloride, *IP3* inositol trisphosphate, *NFATc1* nuclear factor of activated T-cells cytoplasmic-1.

On the molecular level, Fig. 2 shows that HgCl_2_ has markedly boosted the protein contents of (A) IP3 and (B) NFATc1 to 13 and 14 folds, respectively, compared to the control group. Treatment with AEA, on the other hand, noticeably quelled the contents of IP3 and NFATc1 by 84 and 79%, respectively, compared to HgCl_2_ group.

### AEA obliterates the HMGB-1/RAGE/NF-κB hub and PPAR-γ in HgCl_2_-induced AKI

**Figure 3:**
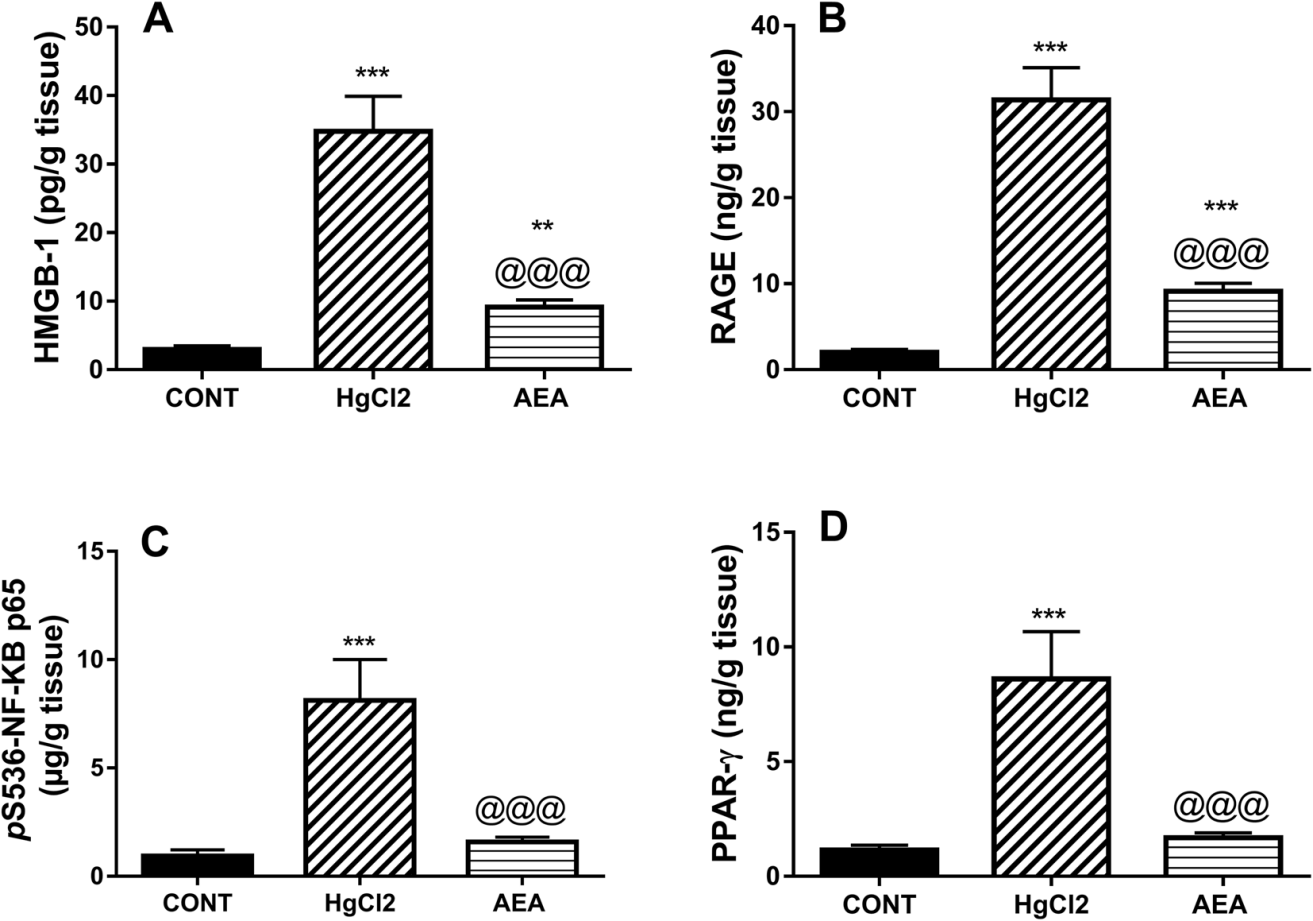
Effect of AEA on renal contents of (**A**) HMGB-1, (**B**) RAGE, (**C**) *p*S536-NF-κB p65, and (**D**) PPAR-γ in HgCl_2_-induced AKI in rats. Data are expressed as the mean of 6 rats ± SD. **P < 0.01 and ***P < 0.001 compared with CONT and ^@@@^P < 0.001 compared with HgCl_2_ groups using one-way ANOVA followed by Tukey's Multiple Comparison post hoc test. *AEA* anandamide, *AKI* acute kidney injury, *CONT* control, *HgCl*_*2*_ mercuric chloride, *HMGB-1* high mobility group box, *pS536-NF-κB p65* nuclear factor-κB p65 phosphorylated at serine 536, *PPAR-γ* peroxisome proliferator-activated receptor gamma, *RAGE* receptor for advanced glycation end products.

The HgCl_2_-induced inflammatory cascade (Fig. 3) was verified by the 12 fold-increase in the content of (A) HMGB-1, its receptor (B) RAGE (16 folds), and the transcription factor (C) *p*S536-NF-κB p65 (9 folds); surprisingly the nuclear ligand-dependent transcriptional factor (D) PPAR-γ (7 folds) was enhanced relative to the control animals*.* On the contrary, AEA curtailed the elevated parameters by 74, 71, 82, and 83%, respectively, as compared to the toxic mercurial salt.

### AEA inhibits caspase-1-mediated activation of IL-18 in HgCl_2_-induced AKI

**Figure 4:**
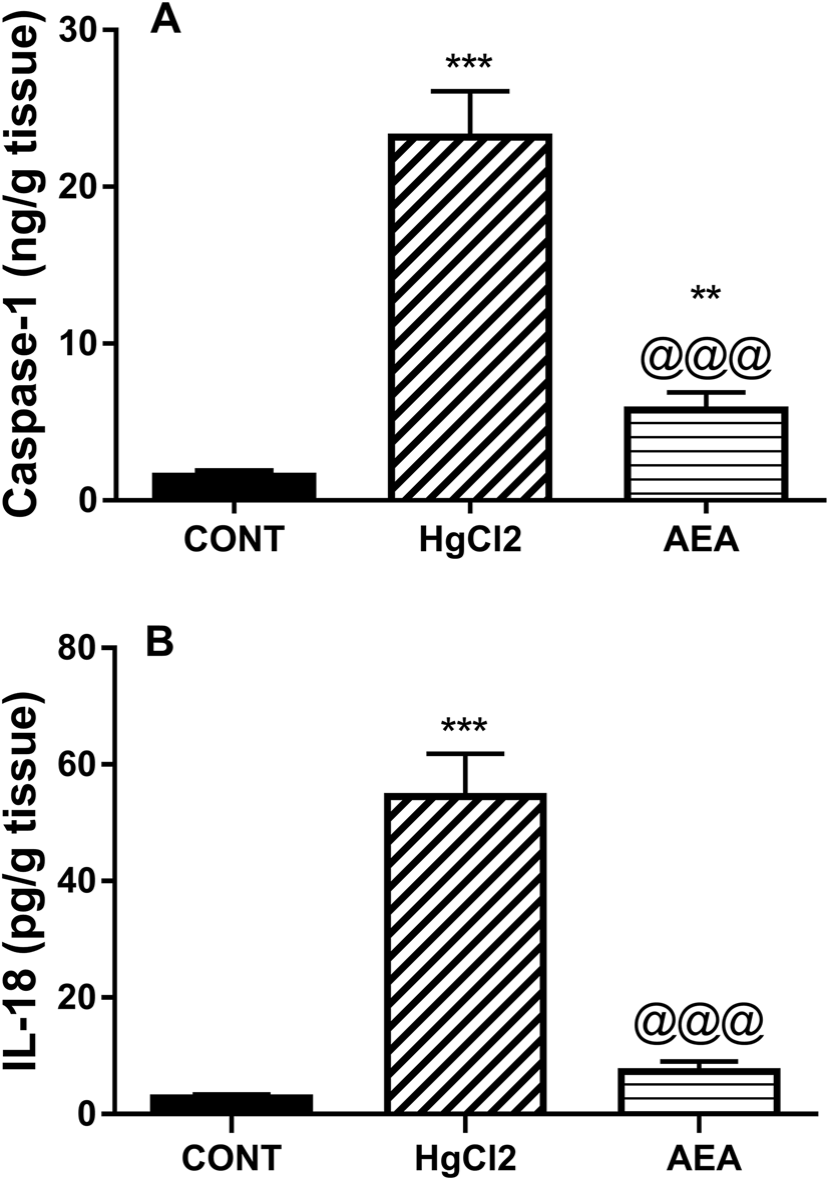
Effect of AEA on renal content of (**A**) caspase-1 and serum level (**B**) IL-18 in HgCl_2_-induced AKI in rats. Data are expressed as the mean of 6 rats ± SD. **P < 0.01 and ***P < 0.001 compared with CONT and ^@@@^P < 0.001 compared with HgCl_2_ groups using one-way ANOVA followed by Tukey's Multiple Comparison post hoc test. *AEA* anandamide, *AKI* acute kidney injury, *CONT* control, *HgCl*_*2*_ mercuric chloride, *IL-18* interleukin 18.

To further verify its injurious effect, Fig. 4 shows that HgCl_2_ bolstered the renal content of (A) caspase-1 (15 folds) and serum level of (B) IL-18 (20 folds), as compared to the control rats. However, treatment with AEA sharply suppressed caspase-1 by 75% and IL-18 by 87% compared to HgCl_2_-exposed rats.

### AEA corrects the redox imbalance and apoptotic cell death mediated by HgCl_2_-induced AKI

**Figure 5:**
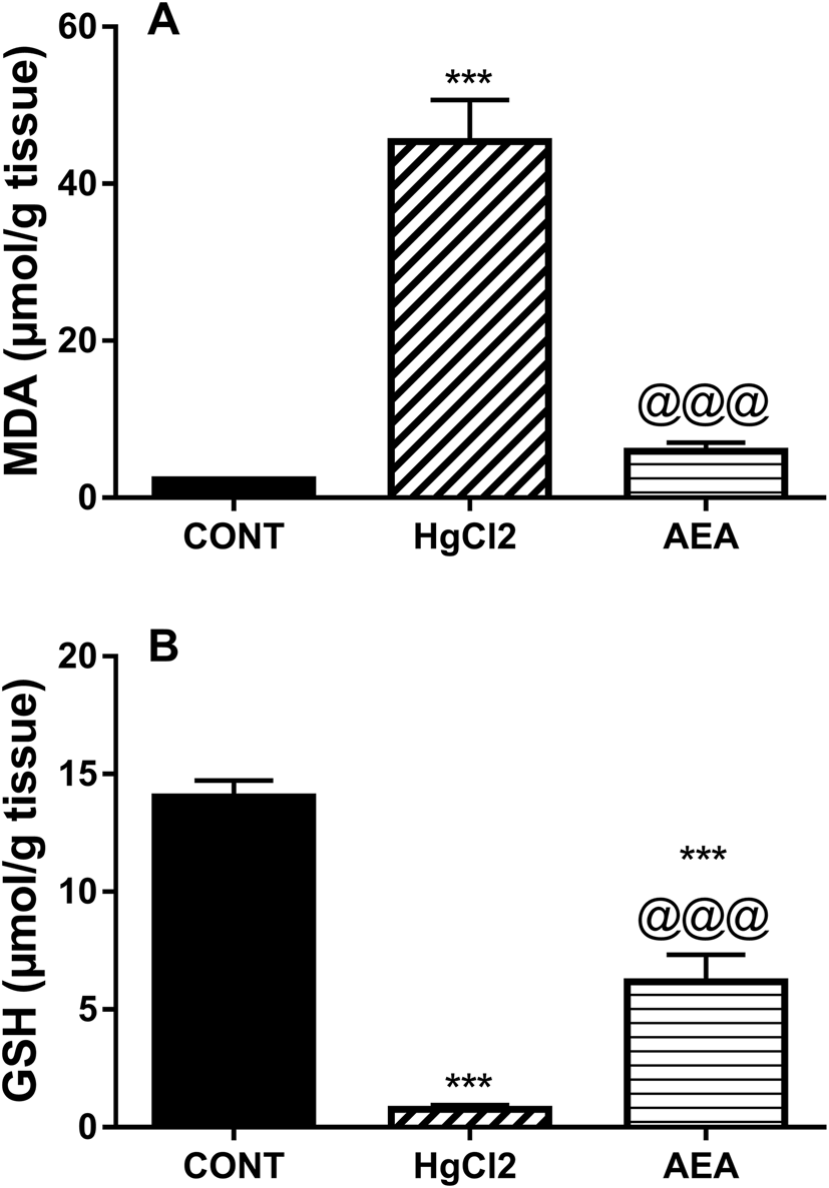
Effect of AEA on renal contents of (**A**) MDA and (**B**) GSH in HgCl_2_-induced AKI in rats. Data are expressed as the mean of 6 rats ± SD. ***P < 0.001 compared with CONT and ^@@@^P < 0.001 compared with HgCl_2_ groups using one-way ANOVA followed by Tukey's Multiple Comparison post hoc test. *AEA* anandamide, *AKI* acute kidney injury, *CONT* control, *GSH* glutathione, *HgCl*_*2*_ mercuric chloride, *MDA* malondialdehyde.

**Figure 6:**
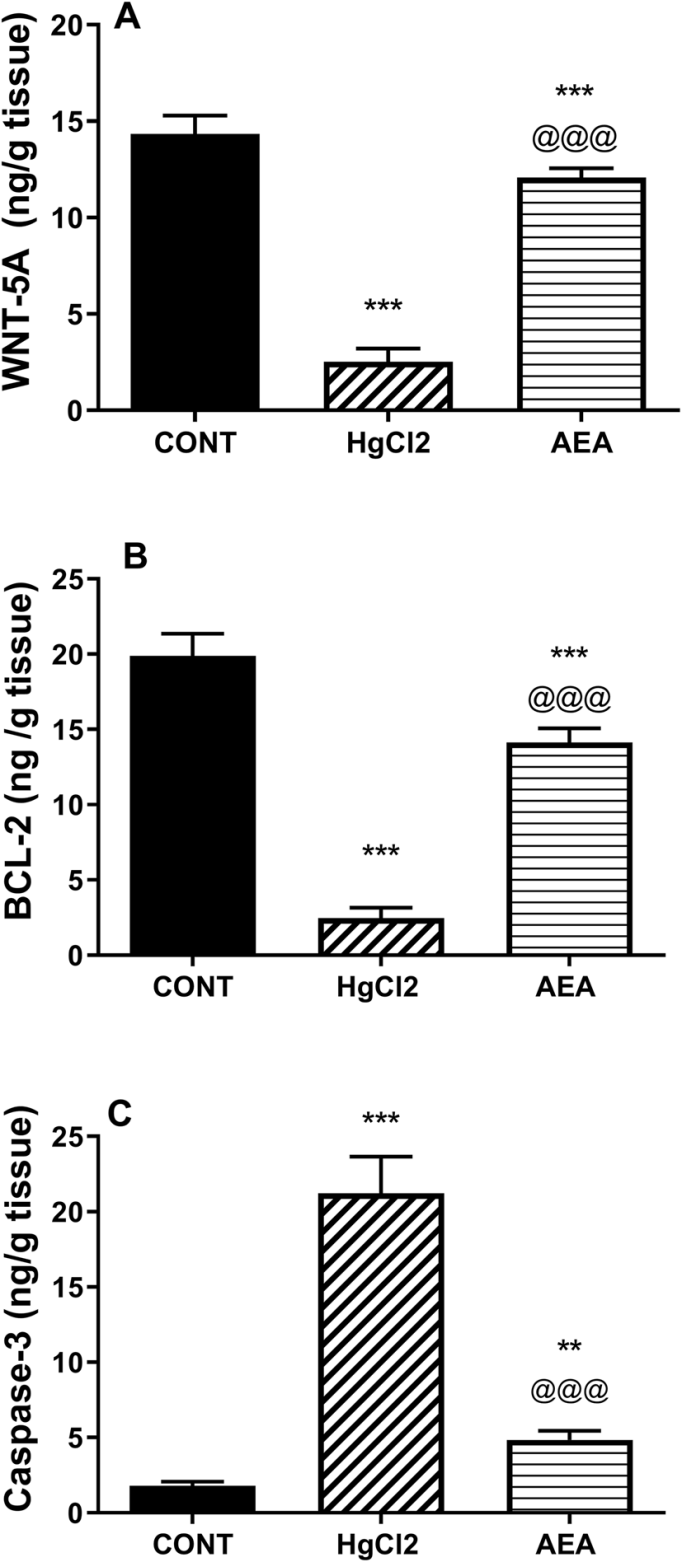
Effect of AEA on renal contents of (**A**) WNT-5A and (**B**) BCL-2, as well as (**C**) caspase-3 activity in HgCl_2_-induced AKI in rats. Data are expressed as the mean of 6 rats ± SD. **P < 0.01 and ***P < 0.001 compared with CONT and ^@@@^P < 0.001 compared with HgCl_2_ groups using one-way ANOVA followed by Tukey's Multiple Comparison post hoc test. *AEA* anandamide, *AKI* acute kidney injury, *BCL-2* B-cell lymphoma-2, *CONT* control, *HgCl*_*2*_ mercuric chloride.

Apart from its inflammatory effect, HgCl_2_ increased also oxidative stress (Fig. 5) signified by the 20-fold increase in (A) MDA content and the depletion of the non-enzymatic defense molecule (B) GSH to 5% compared to the control value. These effects were associated with an apoptotic cascade (Fig. 6), where HgCl_2_ depleted (A) WNT-5A to reach 18% only and decreased the anti-apoptotic marker (B) BCL-2 by 88% but heightened the apoptotic enzyme (C) caspase-3 (12 folds) as compared to the normal group. On the other hand, AEA antioxidant effect was validated by the significant decrease in MDA and the increased GSH renal contents, as compared to the insult-related values. Furthermore, the AEA anti-apoptotic character was reflected by the decrease in caspase-3 by 77% but the upsurge in BCL-2 content and the 5-fold elevation in WNT-5A compared to the HgCl_2_ values.

### AEA corrects the histopathological alterations induced by HgCl_2_ in the rat kidney

**Figure 7:**
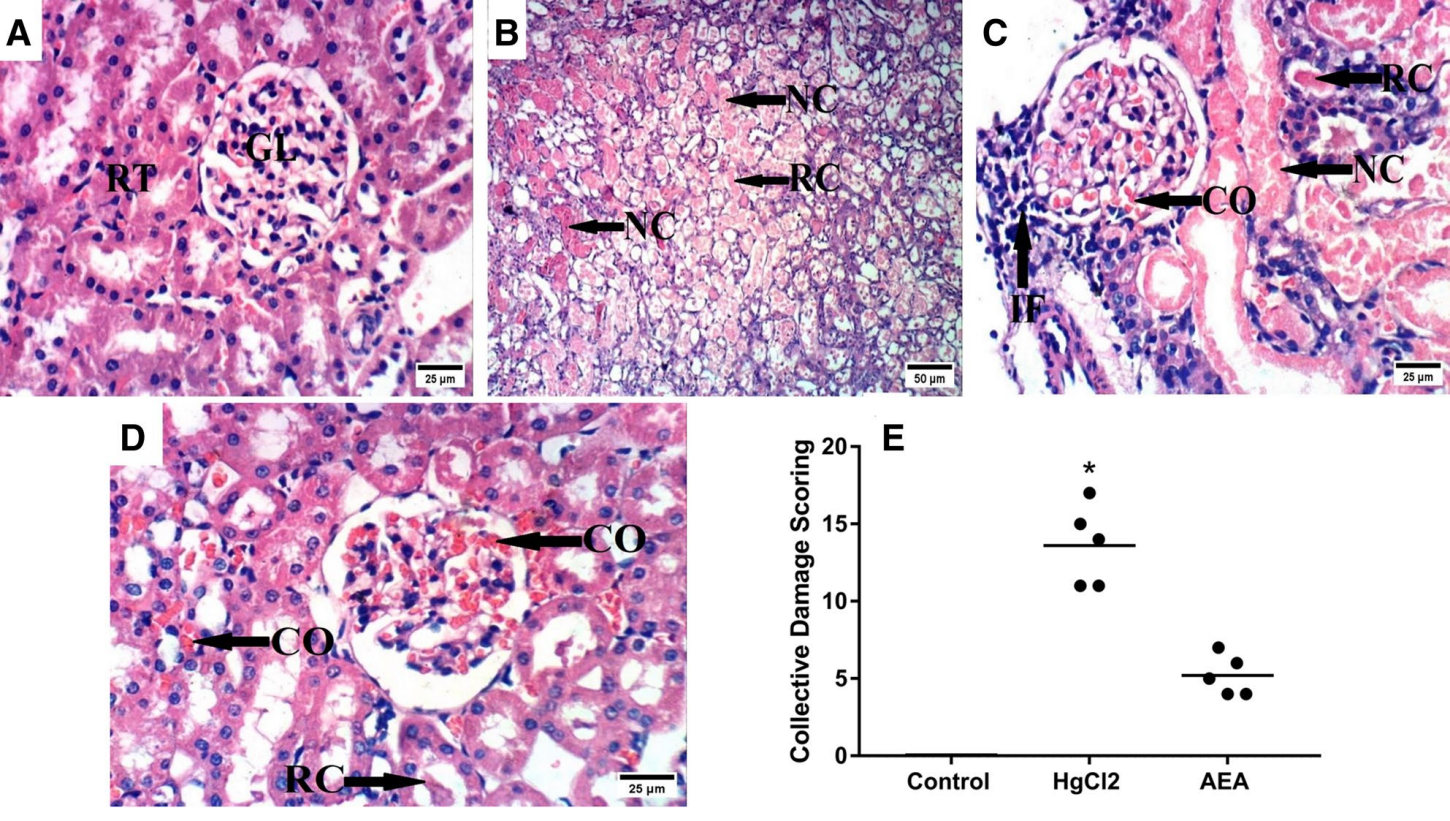
Effect of AEA on HgCl_2_-induced histopathology alterations. Microscopically, the section of the (**A**) normal control group reveals the normal histological architecture of renal parenchyma (normal renal tubules and glomeruli). In contrast, the sections of the (**B**,**C**) HgCl_2_-untreated rats show notable histopathological damage characterized by congestion of glomerular tufts, marked coagulative necrosis of epithelial lining renal tubules, renal cast in the lumen of renal tubules, and inflammatory cells infiltration. On the other hand, the section of the (**D**) AEA-treated group shows an improved picture with milder congestion of glomerular tufts and renal blood vessels as well as renal cast in the lumen of some renal tubules. All black arrows point to the altered structure in the section (scale bar = 25;50). Panel (**E**) illustrates the collective kidney damage score scatter blot; and data were analyzed using the Kruskal–Wallis test followed by Dunn’s post hoc test. Values were calculated from 5 fields of n = 3 rats/group; as compared with *P < 0.05 compared with CONT group. *AEA* anandamide, *HgCl*_*2*_ mercuric chloride.

Figure 7 demonstrates the kidney photomicrograph sections (H&E; scale bar 25 and 50 µm). The sections of (B and C) HgCl_2_ untreated group show notable histopathological damage characterized by congestion of glomerular tufts, marked coagulative necrosis of epithelial lining renal tubules, renal cast in the lumen of renal tubules, and inflammatory cells infiltration compared to the section of (A) the control group, which depicts the normal architecture of renal parenchyma. However, an improved picture is spotted in section of (D) the AEA-treated group showing less epithelial coagulative necrosis of renal tubules compared to the insult. Panel E represents their effect on the collective scores of the lesions, whereas the individual lesion scores are presented in Table 1.

**Table 1:** Effect of AEA on individual kidney damage scores induced by HgCl_2_ in rats.

Groups	Parameters			
	CO	NC	RC	IF
CONT	0 (0–0)	0 (0–0)	0 (0–0)	0 (0–0)
HgCl_2_	3 (2–4)*	5 (4–5)*	4 (3–5)*	2 (2–3)*
AEA	3 (2–3)*	1 (1–2)	1 (1–2)	0 (0–0)^@^

Data are presented as median (min–max) and analyzed using the Kruskal–Wallis test followed by Dunn’s post hoc test. Values were calculated from 5 fields of n = 3 rats/group. Relative to the CONT (*P < 0.05) and HgCl_2_ (^@^P < 0.05) groups. *AEA* anandamide, *CO* congestion of glomerular tufts and renal blood vessels, *HgCl*_*2*_ mercuric chloride, *IF* inflammatory cells infiltration, *NC* necrosis of tubular epithelium, *RC* renal cast.

## Discussion

The current study is the first to address the renoprotective effect of AEA against HgCl_2_-induced AKI evidenced by the improved early (cystatin C/ΚIM-1/IL-18) and late (creatinine/BUN) kidney injury diagnostic markers. The ability of AEA to suppress the IP3/NFATc1 axis offers one mechanism against the HgCl_2_-induced cytotoxicity. Besides, the anti-inflammatory potential of AEA was evidenced by the inhibition of the HMGB-1/RAGE hub and its downstream NF-κB p65, which was associated with a decrease in the caspase-1/IL-18 cue, despite the unpredicted decrease of the transcription factor PPAR-γ that was sharply boosted by HgCl_2_. Additionally, AEA pinned down its antioxidant capacity by suppressing lipid peroxidation and augmenting the defense molecule GSH. By hampering the heavy metal-mediated decrease of WNT-5A/BCL-2 and increase in caspase-3, AEA promoted cell survival and reduced apoptosis in the kidney.

Our results showed that HgCl_2_ boosted the IP3/NFATc1 axis, whereas treatment with AEA has abated it. HgCl_2_-induced AKI is known to be mediated partly by disturbing Ca^2+^ homeostasis to increase its intracellular concentration and hence renal cytotoxicity, an effect that was mediated by ER injury^5^, mitochondrial dysfunction, and activation of PLC signal^6^. The current results offer a new mechanism by stimulating the IP3/NFATc1. Indeed, both IP3 and Hg have been shown to stimulate Ca^2+^ release from endogenous stores by activating the IP3R, which in turn activates the Ca^2+^-dependent phosphatase calcineurin^42,43^. The latter dephosphorylates/activates NFATc1 resulting in its nuclear accumulation and transcriptional activity^42,44^. Of note, previous studies have tethered activated NFATc1 to kidney injury, leading to proteinuria, glomerulosclerosis, and I/R-induced AKI^45–47^, verities that brace the present injurious role of IP3/NFATc1 in the HgCl_2_-induced AKI. A previous clinical study also showed that the use of cyclosporine A, an inhibitor of calcineurin, ameliorated idiopathic membranous nephropathy^48^, as well as proteinuria^49^. Thus, the inhibition of IP3/NFATc1 in the AEA-treated group offers one mechanism for its renoprotection, which may be attributed to a decrease in Ca^2+^, a notion that can be clarified by an earlier study^50^ using activated murine bone marrow-derived mast cells. These co-authors provided evidence for the important role of the AEA-induced CB2R/GPR55 heterodimer in limiting the rise of intracellular Ca^2+^ to suppress mast cell degranulation that was not CB1R dependent.

The current model depleted WNT-5A to be markedly increased by the AEA administration. WNT-5A is an upstream protein of multiple signaling pathways including the non-canonical WNT cascade to increase intracellular Ca^2+^ partly by stimulating the IP3/NFATc1 axis to trigger cytotoxicity^8,18^. However, as proven herein, its beneficial effect is not related to the IP3/NFATc1 cue but may rely on its anti-inflammatory capacity, a notion that concurs with earlier studies pointing to its anti-inflammatory capacity^19,20,51^.

Additionally, activated WNT-5A can play a role in reducing cell demise by regulating cell survival and resistance to apoptosis in different cell lines as previously documented^52,53^. The current results showed that the AEA-treated group displayed anti-apoptotic and cell survival potentials evidenced by increasing the anti-apoptotic molecule BCL-2 and inhibiting caspase-3, besides augmenting WNT-5A, which was reported to also mediate the formation of BCL-2^54^. The reduction of cystatin C and KIM-1 is another culprit for AEA-mediated cell survival, where cystatin C has been reported to downregulate the expression of BCL-2^55^ and KIM-1, which was boosted after HgCl_2_ injury here and hitherto^56^ was expressed in all apoptotic tubules in a chronic cadmium nephrotoxicity model^57^ facilitating apoptotic epithelial cell phagocytosis^58^.

The HgCl_2_-mediates injury is associated with the overproduction of reactive oxygen species (ROS) and the overwhelming of the defense system^3,4^. Thus, the antioxidant aptitude of AEA verified by curtailing the increased lipid peroxidation and elevating the renal content of the defense molecule GSH proffers a further mechanism for its ability to protect against HgCl_2_-induced AKI. The antioxidant effect of AEA may be partly linked to the suppressed IP3/NFATc1 axis, where the HgCl_2_-induced oxidative stress^59^ was especially linked to hydrogen peroxide (H_2_O_2_), which partakes in the generation of IP3^60^. Moreover, it was reported that the transcription factor NFATc1, among others, participates in the transcriptional regulation of NADPH oxidase, one of the crucial oxidative enzymes^61^. Moreover, the restored WNT-5A can play a role in the antioxidant profile of AEA, where Lin et al.^62^ recounted that exogenous administration of the antioxidant enzyme superoxide dismutase increased WNT-5A to protect against in a diabetic nephropathy model.

Scarce data regarding the antioxidant effect of AEA are available; in one study AEA suppressed intracellular ROS and augmented GSH in H_2_O_2_-induced HT22 neuronal cells^63^. The latter co-workers owed the antioxidant response of AEA to the activation of CB1R, and recently, the selective CB2R agonists 1-phenylisatin and HU-308 have attenuated the cisplatin-induced renal oxidative stress^25,35^. Moreover, in an experimental myocardial infarction, Wang et al*.*^64^ stated that stimulation of CB2R facilitates the antioxidant machinery to induce cellular antioxidant genes that control GSH synthesis and regeneration^65^. Moreover, Gallelli et al*.*^66^ provided an overview of the role of CB and non-CB receptors for AEA in mediating its antioxidant effect not only CB2R.

In addition to the corrected redox balance, AEA confirmed its anti-inflammatory potential by inhibiting the inflammatory transcription factor NF-κB to support the results of a previous in vitro model^67^. A high degree of complexity characterizes the interaction between ROS and NF-κB, where, in a vicious cycle, the latter triggers the production of the superoxide anion radical responsible for the activation of the ROS/NF-κB cue^11^. Accordingly, by its antioxidant/anti-inflammatory properties, AEA interjects the ROS/NF-κB vicious cycle, affording further clarification of its ability to suppress IP3/NFATc1 signaling.

Besides the induction of ROS, NF-κB is responsible for the expression of an array of pro-inflammatory mediators, including the renal injury marker IL-18^12^, a verity that is reflected herein and associated by inflammatory cell infiltration as shown in the microphotographs of HgCl_2_ exposed kidney. Along with the boosted IL-18, HgCl_2_ heightened its activator caspase-1 to suggest a possible role for inflammasome signaling in the HgCl_2_-induced AKI, a point that warrants investigation. Treatment with AEA, on the other hand, has sharply reduced both IL-18 and its activator, caspase-1, to match the findings of Li et al*.*^16^, who showed that AEA inhibited the NLRP3 inflammasome and its downstream axis caspase-1/IL-18. The endocannabinoid also reduced the renal inflammatory cell infiltration to corroborate the decline in this inflammatory cytokine.

The HMGB-1/RAGE hub was also bolstered by HgCl_2_ to be markedly suppressed by AEA. When HMGB-1, an endogenous danger-associated molecular pattern molecule, is released from the nucleus in a ROS-dependent manner^68^, it reactivates NF-κB upon binding to its receptor RAGE in a hostile intercalating loop^69^. Hence, the aptitude of AEA to oppose the inflammatory effect of HgCl_2_ and to inhibit the HMGB-1/RAGE cue, documented herein for the first time, pins down the anti-inflammatory capacity of AEA, besides its antioxidant effect. It is worth mentioning that the current antioxidant and earlier reported anti-TNF-α effects of AEA^70^ may be responsible also for the anti-apoptotic effect of AEA since they can inhibit caspase-3 by both intrinsic and extrinsic death pathways, respectively.

The third transcription factor investigated herein is PPAR-γ, which was unexpectedly upregulated in the HgCl_2_ group, a response that might be compensatory since the activation of PPAR-γ is well documented to antagonize NF-κB, ROS, and inflammation^71^. This assumption could be braced by earlier findings in spontaneously hypertensive rat aorta and mesenteric resistance arteries^72^. On the other hand, the AEA-associated decrease in PPAR-γ can be explained by the ability of the endocannabinoid to quench ROS and inhibit inflammation, as seen here, which no longer necessitates the upregulation of PPAR-γ.

To this end, we have reported the nephroprotective role of AEA, which amended HgCl_2_-induced AKI by multiple intersecting mechanisms. AEA reduced inflammation, oxidative stress, and apoptosis through the activation of the WNT-5A/BCL-2 survival pathway and inhibiting the IP-3/NFATc1, HMGB-1/RAGE/NF-κB, and caspase-1/IL-18 cues.

## Data Availability

All data generated or analyzed during this study are included in this article.

## Abbreviations

AEA: Anandamide
ANOVA: Analysis of variance
BCL-2: B-cell lymphoma-2
CB2R: Cannabinoid 2 receptor
BUN: Blood urea nitrogen
CONT: Control
DAG: Diacylglycerol
HMGB1: High mobility group box protein-1
AM630: 6-Iodopravadoline
PIP2: Phosphatidylinositol 4,5-bisphosphate
IP3: Inositol trisphosphate
KIM-1: Kidney injury molecule-1
LPS: Lipopolysaccharide
IL-18: Interleukin 18
MDA: Malondialdehyde
HgCl_2_: Mercuric chloride
NF-κB: Nuclear factor-κB p65
NFATc1: Nuclear factor of activated T-cells 1
RAGE: Receptor of glycated end products
PPAR-γ: Peroxisome proliferator-activated receptor-γ
